# Early signs of pneumoconiosis in a dental technician in Italy: a case report

**DOI:** 10.1186/s12890-021-01721-1

**Published:** 2021-11-07

**Authors:** Mara Maria Tiraboschi, Emma Sala, Matteo Ferroni, Andrea Tironi, Andrea Borghesi, Maria Enrica Gilberti, Paolo Ceruti, Emanuele Sansone, Giuseppe De Palma

**Affiliations:** 1 Department of Medical and Surgical Specialties, Radiological Sciences, and Public Health, Unit of Occupational Health and Industrial Hygiene, University of Brescia, Brescia, Italy; 2 Occupational Health, Occupational Hygiene, Toxicology and Prevention Unit, University Hospital “Spedali Civili Di Brescia”, Brescia, Italy; 3 CNR-Institute of Microelectronics and Microsystems, Bologna, Italy; 4 Department of Civil, Environmental, Architectural Engineering and Mathematics, University of Brescia, Brescia, Italy; 5 Pathology Department, University Hospital “Spedali Civili Di Brescia”, Brescia, Italy; 6 Department of Medical and Surgical Specialties, Radiological Sciences, and Public Health, Unit of Radiological Sciences, University of Brescia, Brescia, Italy; 7 Pulmonology Department, University Hospital “Spedali Civili Di Brescia”, Brescia, Italy

**Keywords:** Dental technician, Metals, Pneumoconiosis, Occupational disease, Case report

## Abstract

**Background:**

Dental technicians are at high risk of pneumoconiosis, usually driven by inhalation of mixed dusts, including metals. An etiological diagnosis is not easy to be performed, particularly in advanced stages.

**Case presentation:**

We describe the case of an early pneumoconiosis occurring in a 47-year-old dental technician who developed respiratory symptoms shortly after beginning work. She described the work environment as dusty and lacking relevant primary prevention tools. A chest CT showed multiple peripheral pseudonodular lesions in both lower lobes; bronchoalveolar lavage and bronchial aspirate evidenced numerous macrophages with reflective metal bodies included into the cytoplasm, that at scanning electron microscopy coupled to Energy Dispersive X-Ray Analysis resulted Zirconium and Aluminum, whereas Tungsten (W) was localized outside cells. End of shift urinary concentrations of W were substantially raised as compared to pre-shift (1.1 vs. 0.2 µg/L).

**Conclusions:**

We concluded for diagnosis of early work-related pneumoconiosis due to abnormal occupational exposure to metals. The case demonstrates the need also for dental professionals to comply with industrial hygiene standards and to be monitored by occupational health physicians.

## Background

Dental technicians are exposed to pneumotoxic elements, including crystalline silica and hard metal alloys that can lead to pneumoconiosis [1–4]. Recently, a cluster of 9 cases of idiopathic pulmonary fibrosis (IPF) among dentists and other dental professionals has been reported [5]. An etiological diagnosis is not easy to be performed, particularly if formulated at an advanced stage. We describe an early pneumoconiosis occurring in a dental technician who developed early respiratory symptoms shortly after beginning work.

## Case presentation

**Fig. 1:**
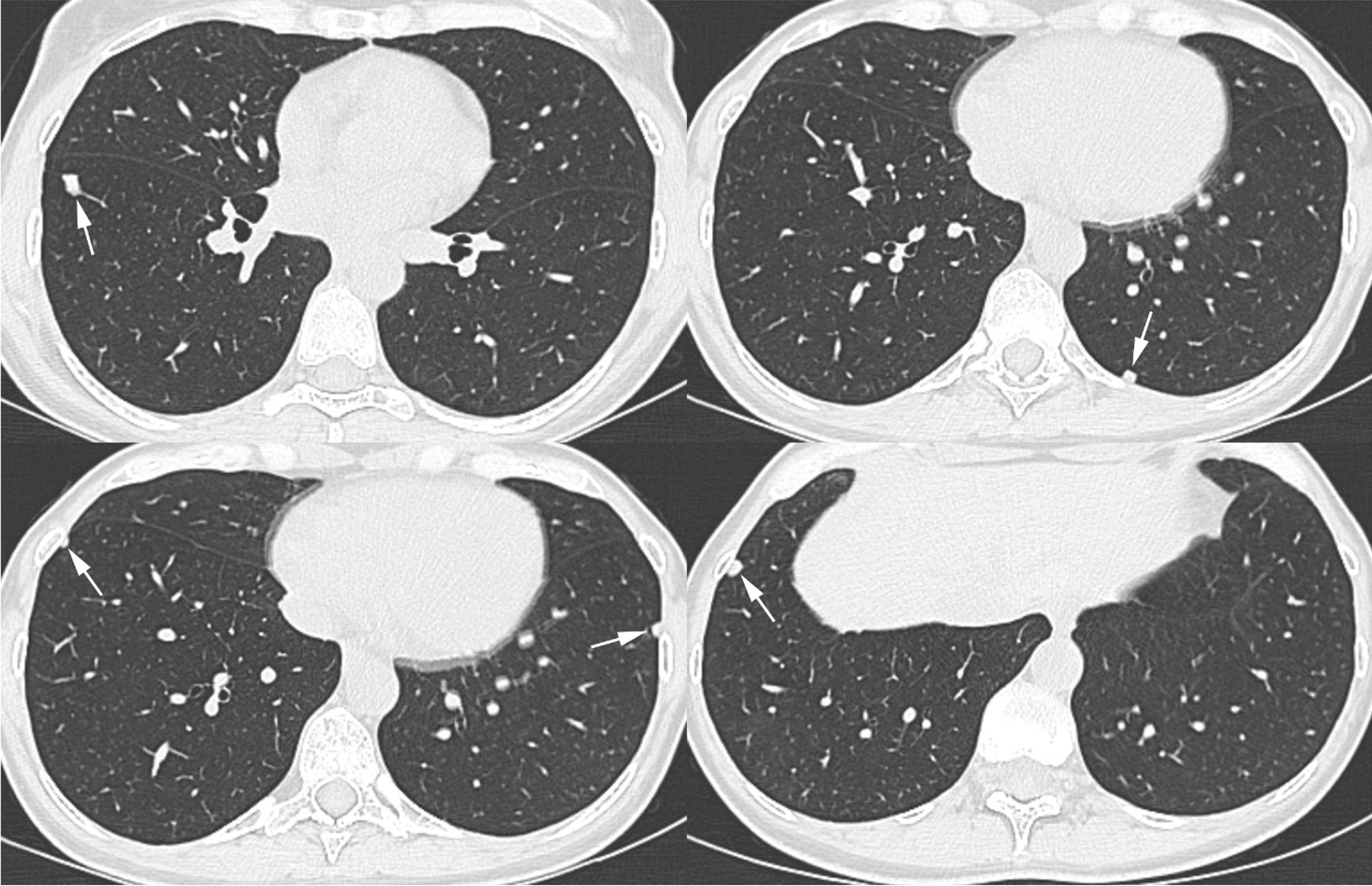
Axial thin-section CT images with lung window setting show multiple peripheral pulmonary nodules (arrows) in both lower lobes

On 27th July 2020, a 47-year-old woman, working as a ceramic dental technician since November 2018, required a medical examination at our Occupational Health Dept., as in the previous months she was affected by dry and irritating cough, especially related to intense work activity. She also suffered some episodes of low-grade fever and fatigue and dyspnea on efforts. In March 2020, chest CT showed lung nodules characterized by net margins and oval shape, in peripheral or subpleural site, especially in the inferior lobes. The biggest nodule, 9 mm, with polygonal shape, was in the anterior basal segment of the righ inferior lobe (Fig.1).

Pulmonary function tests were in the normality range and revealed a normal diffusing capacity, albeit at the lower limits of the normal (DLCO/VA 73%); autoimmunity markers were in the normal range. Then, she underwent fibro-bronchoscopy with bronchoalveolar lavage (BAL) and bronchial aspirate (BAS). BAL differential cell count showed macrophages (79%), lymphocytes (18%), neutrophil granulocytes (3%), and a CD4/CD8 lymphocyte ratio of 7.8. In both BAL and BAS, several macrophages with numerous intracytoplasmic exogenous metallic material and reflective dust were seen at optical microscopy. Owing to the SARS-CoV2 pandemic, she was absent from workplace from March to August 2020 and in such period her symptoms ameliorated. At work, the woman was involved in milling and polishing monolithic zirconia structures, then layered with ceramic. She described workplace as dusty, lacking aspiration hoods hence she was concerned about workplace safety. She used respiratory personal protective equipment (PPE) inconstantly.

**Fig. 2:**
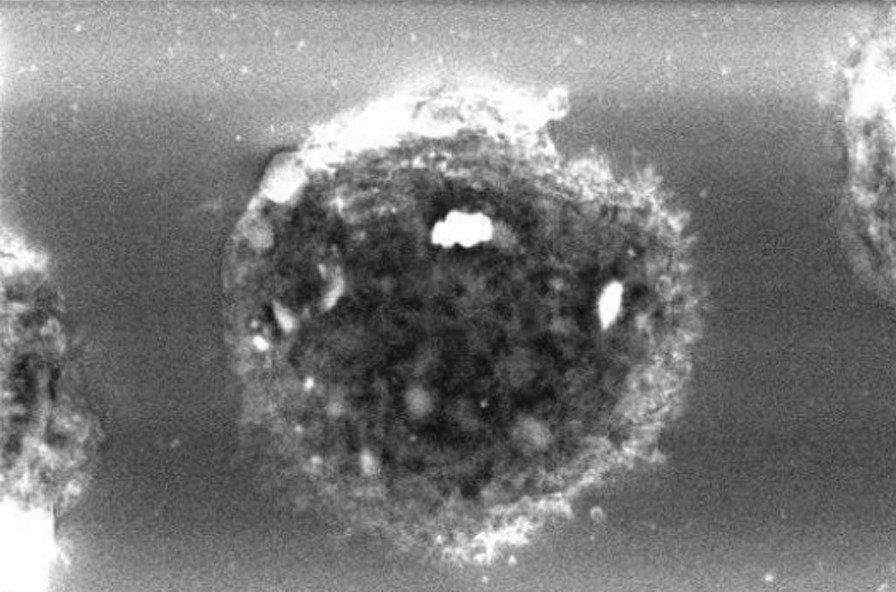
An image taken with scanning electron microscopy (SEM) coupled to Energy Dispersive X-Ray Analysis (EDX) showing numerous inorganic particles, containing Zr and Al within the macrophages

**Fig. 3:**
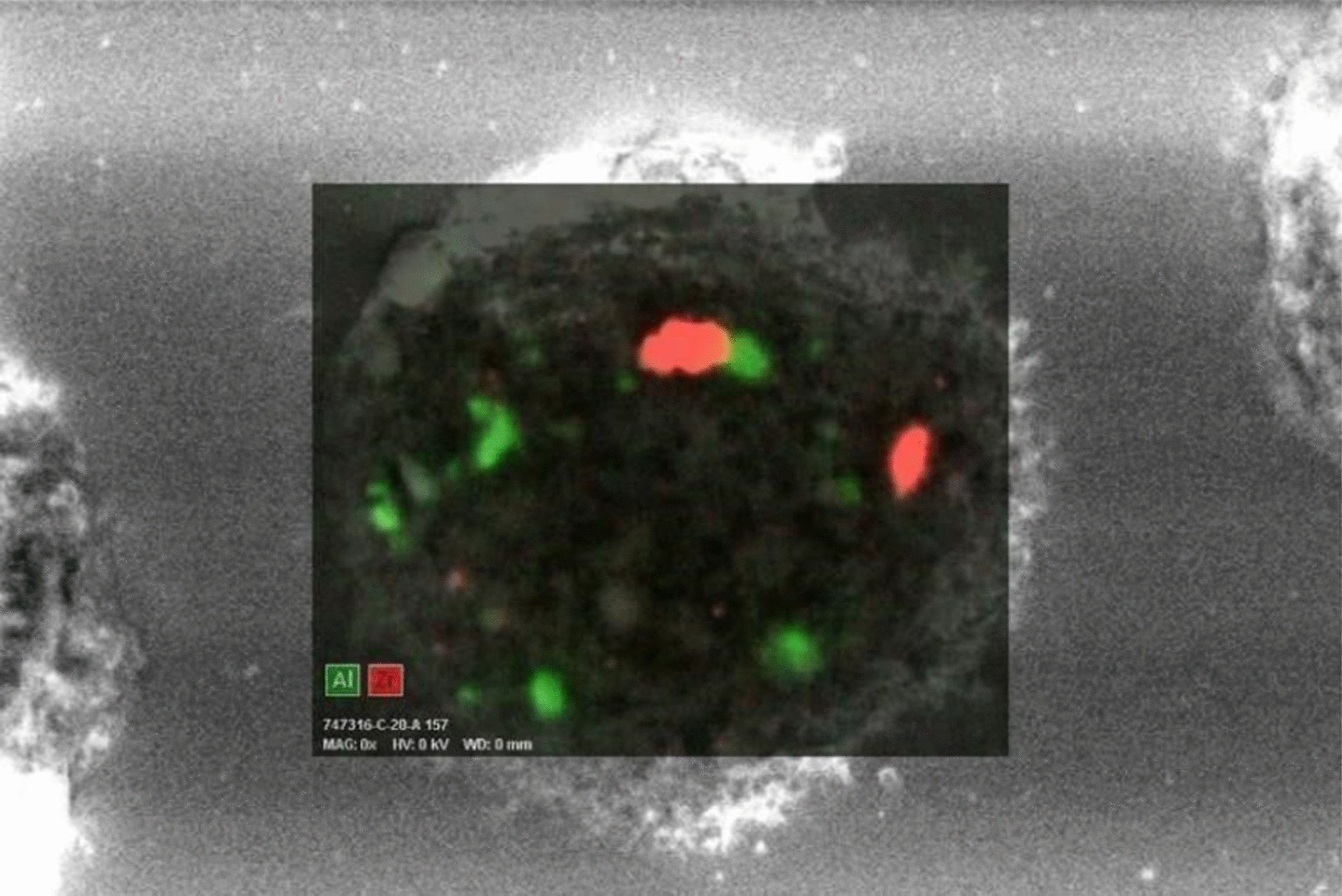
A detail of the previous image: the green areas show the zones with the biggest concentration of Al, the red areas are the ones with the major concentration of Zr

We examined three samples of dust settled nearby her workstation by inductively coupled mass spectrometry (ICP-MS) [6]. Among others, we detected average Cobalt (Co), Tungsten (W), Zirconium (Zr) and Yttrium (Y), a component of dental ceramic) at concentrations of 45, 60, 96 and 176 µg/g, respectively. Apart from Co, the same elements could be detected, always by ICP-MS, on a paraffin-embedded BAS sample. On BAL cytocentrifugated slides, scanning electron microscopy (SEM) coupled to Energy Dispersive X-Ray Analysis (EDX) showed numerous inorganic particles, containing Zr and Aluminum (Al), the latter probably as oxide, within the macrophages (Figs. 2, 3). In addition, a signal attributed to W was detected but not localized in a specific image detail. About a month after return at work, we investigated metal concentrations in urine (U) and in exhaled breath condensate (EBC), collected either at the beginning and at the end of a workweek, by ICP-MS. We could demonstrate a 6 times weekly increase of urinary concentrations of W (0.18 µg/L vs. 1.1 µg/L). We concluded for a diagnosis of early work-related pneumoconiosis due to abnormal occupational exposure to metals. We advised periodical chest CT and pulmonologist monitoring (the next after 6 months), along with the prescription to strictly wear respiratory personal protective equipment (PPE) at work.

## Discussion and conclusions

Dental technicians are at high risk of pneumoconiosis (prevalence rate 4.5–23.6% after a mean exposure duration of 12.8–28.4 years) [4, 7–10, 15], usually driven by inhalation of mixed dusts, including crystalline silica and hard metals, mostly chromium-cobalt-molybdenum alloys [3, 11, 12].

Usually, diagnosis is formulated after several years of exposure. The CT or RX patterns consist of micronodular and reticular lesions usually in upper-middle lung lobes and restrictive and/or obstructive lung function impairment [13–15]. Our case developed pulmonary symptoms early after work beginning. According to occupational history and analytical results, she was heavily exposed to mixed dusts, including W, Al oxide and Zr, both the latter known to be pneumotoxic [16–18]. Al oxide can elicit sarcoid-like or berylliosis-like lung granulomatosis, accompanied by T-helper lymphocyte alveolitis [19], whereas Zr can induce a "benign" pneumoconiosis with little or no fibrosis [16]. W alone is not toxic but, if associated to Co, it may give rise to the very insidious hard metal lung disease [20]. In such event, Co is very soluble and hence often its detection in lung tissue samples fails. In the presented case, the high exposure levels to mixed metal dusts, coupled to some yet unrecognized individual susceptibility trait, may have determined the accelerated development of lung damage. A main limitation affecting our diagnostic potential was the unavailability of lung tissue. In conclusion, the case demonstrates that dental technicians are at risk of developing lung disease. Prevention in such work sector implies careful risk assessment through airborne and biological monitoring of pneumotoxic metals.

## Data Availability

Data sharing is not applicable to this article as no datasets were generated or analyzed during the current study.

## Abbreviations

Al: Aluminum
BAL: Bronchoalveolar lavage
BAS: Bronchial aspirate
Co: Cobalt
EBC: Exhaled breath condensate
EDX: Energy Dispersive X-Ray Analysis
ICP-MS: Inductively coupled mass spectrometry
IPF: Idiopatic pulmonary fibrosis
PPE: Personal protective equipment
SEM: Scanning electron microscopy
U: Urine
W: Tungsten
Y: Yttrium
Zr: Zirconium
